# Purification and Characterization of Recombinant Human Lysozyme from Eggs of Transgenic Chickens

**DOI:** 10.1371/journal.pone.0146032

**Published:** 2015-12-29

**Authors:** Hanyu Wu, Dainan Cao, Tongxin Liu, Jianmin Zhao, Xiaoxiang Hu, Ning Li

**Affiliations:** 1 State Key Laboratory for Agrobiotechnology, College of Biological Sciences, China Agricultural University, Beijing 100193, P. R. China; 2 Wuxi KGBIO Biotechnology Limited Liability Company, Jiangsu 214145, P. R. China; Wuhan University, CHINA

## Abstract

Transgenic chickens as bioreactors have several advantages, such as the simple establishment procedure, correct glycosylation profile of expressed proteins, etc. Lysozyme is widely used in food industry, livestock farming, and medical field as a replacement of antibiotics because of its antibacterial and complement system-modulating activity. In this study, we used RT-PCR, Western blot, and immunofluorescence to detect the expression of recombinant human lysozyme (rhLY) in the transgenic chicken. We demonstrated that the transgene of rhLY was genetically stable across different generations. We next optimized the purification procedure of rhLY from the transgenic eggs by utilizing two steps of cation-exchange chromatography and one gel-filtration chromatography. About 6 mg rhLY with the purity exceeding 90% was obtained from ten eggs, and the purification efficiency was about 75%. The purified rhLY had similar physicochemical and biological properties in molecular mass and antibacterial activity compared to the commercial human lysozyme. Additionally, both of them exhibited thermal stability at 60°C and tolerated an extensive pH range of 2 to 11. In conclusion, our study proved that the transgenic chickens we have previously generated were genetically stable and suitable for the production of active rhLY. We also provided a pipeline for purifying the recombinant proteins from transgenic eggs, which could be useful for other studies.

## Introduction

Antibiotics added to the diets of poultry and pigs can improve animal productivity, health and meat quality [1]. However, the antibiotic resistance of microorganisms limits the use of antibiotics [2,3]. Lysozyme could substitute some antibiotics to improve intestinal morphology and reduce microbial challenges in the intestine [1,4,5] because of its anti-inflammatory capabilities [1,6]. Lysozyme, also known as muramidase, is a 1,4-β-N-acetylmuramidase that degrades the glycosidic bonds in the peptidoglycan of bacterial cell walls [7]. It is widespread in tears, saliva, blood serum, human and cow milk, and avian egg whites [8,9]. A crucial capacity of lysozyme is to protect against infections of bacteria, viruses, and fungi [10–12]. Thus, it could be used as a preservative in the food industry. Besides, lysozyme was reported to modulate the complement system as an immune regulator [13,14]. Therefore, food industry, husbandry, and medical field demand a large amount of lysozyme.

According to the differences in the amino acid sequences and protein tertiary structures, lysozymes are classified into C-type, T4-type, and G-type [15–17]. Both chicken egg lysozyme (cLY) and human lysozyme (hLY) belong to C-type. A previous study showed that there were no detectable cross reactions between cLY and hLY [18]. Although cLY is easily obtained from egg whites, hLY displays a three-fold greater antibacterial activity and higher thermal stability [19] due to the differences in cationic residues [20] and tridimensional structures. Additionally, hLY is more effective therapeutically to a wide range of human diseases without producing immunogenicity and side effects [21]. However, the limited source of hLY restricts its use. Thus, we focus on transgenic engineering to produce rhLY. Up to date, expression of rhLY has been reported in many bioreactor systems, including the fungus [22,23], plants [1,24], and animals [25–27]. Compared with other bioreactors, transgenic chickens have several advantages, including shorter generation time, lower cost of cultivation, more beneficial glycosylation profile of target proteins and the lower immunogenicity of the protein product [28,29]. Therefore, transgenic chickens are an effective bioreactor for producing recombinant proteins [28,30–32].

The molecular weight of lysozyme is approximately 14 kDa to 15 kDa and the isoelectric point is about pH 11 [33]. These characteristics of lysozyme make the foundation of its purification methods. At early times, Fleming found that lysozyme could be absorbed by many substances, such as charcoal, cellulose, and porcelain [34]. Wolff found some other matrices could adsorb active lysozymes, and the elution efficiency was affected by pH [35]. In 1985, cation-exchange chromatography was used to separate highly functional lysozyme from egg whites by Lichan and Nakai [36]. In 1999, recombinant equine lysozyme and hLY were isolated from filamentous fungus *Aspergillus niger* by ion exchange method [37]. In 2006, PEG/salt aqueous two-phase system was applied to extract lysozyme from chicken egg whites [38]. Later, Wilken used cation-exchange resin to purify hLY from transgenic rice seeds [21], and Yang used cation-exchange chromatography and gel-filtration chromatography to extract high-purity rhLY from transgenic cattle [39]. These reports prove that the adsorption-elution methods are effective for lysozyme separation, and could produce large quantities of lysozyme. Thus, it opens up new opportunities for fundamental studies of the recombinant proteins.

Our prior study has generated transgenic chickens expressing rhLY in the egg whites [30]. The current work tested the genetic stability of the transgene in the transgenic chickens and analyzed the physicochemical and biological characteristics of rhLY. In addition, we optimized the procedure for purifying rhLY from transgenic egg whites, which could be used for industrial purification of rhLY in the future.

## Materials and Methods

### Ethics statement

The husbandry of experimental animals followed the guidelines and provisions of the Use and Care of Laboratory Animals in China. The animal experiment protocol was approved by the Animal Welfare Committee of China Agricultural University. The permission number is SKLAB-2014-06-01.

### Generation and analysis of G3 and G4 transgenic chickens

We have obtained four G1 transgenic chickens with the hLY gene inserting into four different genomic sites [30]. The chicken A011 with the insertion site on Z chromosome was used to generate G2 heterozygous chickens, and G2 heterozygotes were crossed with non-transgenic chickens to produce G3 transgenic offspring. The same breeding protocol was applied to obtain G4 transgenic offspring. PCR was used to screen transgenic chickens. The template DNA was extracted from the cockscombs of G3 and G4 chicks according to the standard HotSHOT protocol [40]. Primers of hLY-F and hLY-R (S1 Table) were used to amplify the hLY gene. Primers of sex-F and sex-R (S1 Table) were used to determine the sex of the transgenic chickens.

### RT-PCR

Total RNA was isolated from the heart, liver, spleen, lung, kidney, intestine and oviduct of G3 and G4 hens with TRIzol reagent (Invitrogen, USA). RNA extracted from the oviduct of a non-transgenic hen was used as the negative control. Then 4μg RNA was taken to synthesize the first strand of cDNA with M-MLV reverse transcriptase (Promega, USA). Primers used for RT-PCR analysis were shown in S1 Table. Primers of hLY-F and hLY-R were used for the hLY gene amplification to detect its tissue-specific expression while RT-GAPDH-F and RT-GAPDH-R were used to amplify the GAPDH gene that served as the internal control.

### Western blot

The egg whites from G3/G4 transgenic hens or wild-type White Leghorns were pretreated with 4 volumes of ice-cold 50 mM sodium acetate buffer (pH 5.0) for 1 hour at 4°C to remove the bulk of ovomucin in the eggs. During the incubation, the mixture was mixed up and down several times. After the mixture had been centrifuged at 3,000 g for 20 minutes, 2 μl of the supernatant was separated by 15% SDS-PAGE and transferred to PVDF membranes for Western blot analysis. Rabbit monoclonal antibody to hLY (1:1000 dilution; Abcam, EPR2995, UK) and horseradish peroxidase–conjugated goat anti–rabbit IgG (1:10000 dilution; ZSGB-BIO, China) were used to detect rhLY. The commercial hLY (100 ng; Sigma-Aldrich, USA) was used as a positive control. The result was analyzed by Image J to quantify the rhLY in the transgenic eggs. Rabbit polyclonal antibody to cLY (1:1000 dilution; US Biological, L9200-05B, USA) and horseradish peroxidase–conjugated goat anti–rabbit IgG (1:20000 dilution) were used to detect cLY, which was an internal control. The positive control of cLY was the purified commercial cLY (Sigma-Aldrich, USA).

### Immunofluorescence analysis

The magnum portions of the adult hen oviducts were fixed in 4% paraformaldehyde for 24 hours and washed in phosphate buffered saline (PBS) for 30 minutes. After that, the tissue was embedded in paraffin and sectioned into 5 μm slices with a Thermo HM550 system (Thermo, America). After rehydration and antigen retrieval, the slides were blocked in blocking solution (ZSGB-BIO, China) for 1 hour. Then they were incubated with mouse monoclonal anti-Human Lysozyme antibody (1:200 dilution; US Biological, L9200-05J, USA) overnight at 4°C, followed by incubation in the secondary antibody (1:400 dilution; Invitrogen, USA) for 1 hour at room temperature. Samples were visualized and imaged using a Leica DM5500 B microscope (Leica, Germany).

### Sample preparation

The majority of ovomucin fractions were first removed from the egg whites of G3 and G4 eggs (200ml from ten eggs) as aforementioned [28]. After centrifugation, the supernatant (about 1 L) was freeze-dried to obtain the lyophilized powder (20 g). Then the powder was dissolved in 20 mM sodium phosphate buffer, pH 8.5 (300 ml). The sample solution was filtered through 0.45 μm filters before use.

### Purification of rhLY

The ÄKTA purifier 10 system (Amersham Bioscience, NJ) was used to isolate rhLY with a three-step chromatographic procedure. In the first chromatographic step, about 300 ml of sample solution was loaded onto a Hiscreen SP FF prepacked column (GE Healthcare, Sweden), which was equilibrated with 20 mM sodium phosphate buffer (pH 8.5) ahead of time. The bound proteins were eluted with 1 M NaCl in 20 mM sodium phosphate buffer (pH 8.5) to collect the majority of alkaline proteins. The consequent fractions were desalted in 20 mM sodium phosphate buffer (pH 7.0) with 3-kDa-cutoff ultracentrifuge tubes (Millipore, USA), and filtered through 0.22 μm filters. Subsequently, the sample was purified by another cation-exchange chromatography through a Mono S 5/50 GL prepacked column (GE Healthcare, Sweden). The bound proteins were eluted with a linear gradient of 0–1 M NaCl in 20 mM sodium phosphate buffer (pH 7.0) to separate rhLY from cLY. Next, fractions containing rhLY were concentrated using a 3-kDa-cutoff ultracentrifuge tube and further purified by gel filtration chromatography through a Superdex^TM^ 75 10/300 GL prepacked column (GE Healthcare, Sweden) in 50 mM sodium phosphate buffer containing 0.15 M NaCl (pH 7.0). The purified rhLY was desalted and concentrated by the ultracentrifuge tubes. At last it was quantified by BCA Protein Assay Kit (Beyotime, China) and tested by SDS-PAGE and Western blotting.

### Molecular weight and peptide mass fingerprinting analysis of purified rhLY

The purified rhLY was sent to Shanghai Applied Protein Technology Co. Ltd (Shanghai, China) to analyze its molecular weight and peptide mass fingerprinting using 5800 MALDI-TOF/TOF (AB Sciex, America).

### N-terminal sequence analysis of purified rhLY

The N-terminal amino acid sequence of the purified rhLY was analyzed by automatic Edman degradation in Shanghai. The resulting N-terminal sequence of the test was aligned to the GeneBank databases of the National Center for Biotechnology Information (GenBank accession no. NC_000012).

### Antibacterial activity analysis of purified rhLY

*Micrococcus lysodeikticus* (China General Microbiological Culture Collection Center, Beijing) cultivated to mid-log phase (OD600≈0.7) in Luria-Bertani broth was mixed with 15 ml nutrient broth agar and plated in 100 mm dishes. Lysozyme samples were applied to sterile, quantitative filter paper discs (6 mm in diameter), placed on the *M*. *lysodeikticus* plate, and then incubated at 30°C for 24 hours. Inhibition zones were used to determine the antibacterial activity. Sterile water was used as a negative control. The experiment was repeated three times.

The turbidimetric method was conducted according to the lytic assay described by Shugar [41]. *M*. *lysodeikticus* used as the substrate was incubated in Luria-Bertani broth for about 20 hours. Then the bacteria were precipitated by centrifugation and resuspended in PBS (pH 7.18) so that the OD450 of the bacterial suspension was about 0.7. Immediately after the bacterial substrate had been mixed with the tested lysozyme samples (100 μl) or PBS (blank), we recorded OD 450 every 15 seconds over a 3-minute period. The ΔOD 450 per minute was applied as the maximum linear rate. The lytic activity of lysozyme was calculated as: Units/mg Protein = (Units/ml enzyme) / (mg protein/ml enzyme) [39]. All samples were measured at least three times.

### Optimal temperature and pH value analysis for lysozyme’s antibacterial activity

The *M*. *lysodeikticus* substrate was resuspended in PBS (pH 7.18) as aforementioned. The purified rhLY or the commercial hLY were added to the substrate solution, and incubated at 25°C, 40°C, 60°C, and 80°C for 10 minutes, separately. The turbidimetric method described above was used to measure the lysozyme activity and determine the optimal temperature.

Phosphate buffers with different pH values (from 2–12) were used to dilute the *M*. *lysodeikticus* cells and lysozyme samples, respectively. Then they were mixed and incubated at room temperature for 10 minutes. The same turbidimetric method was used to calculate the enzyme activity to obtain the optimal pH value of the purified rhLY and commercial hLY. All the experiments above were repeated at least three times.

### Thermostability analysis

The purified rhLY and commercial hLY were diluted in phosphate buffered saline (pH 7.18) and incubated at 100°C, 80°C, or 60°C for 2, 4, 7, 10, 15, 25, 35, and 45 minutes, separately. Then they were added to the bacterial suspension to determine the lytic activity of the lysozymes with the turbidimetric method described above for three times.

### pH stability analysis

Lysozyme samples were incubated in phosphate buffer of different pH values (from 2–12) for 20 min. *M*. *lysodeikticus* cells suspended in PBS (pH 7.18) were used to determine the pH stability of the lysozymes by the turbidimetric method. The experiment was repeated three times.

## Results

### Generation of G3 and G4 chickens

The G2 heterozygous chickens were crossed with non-transgenic White Leghorn and 139 offspring were obtained. Among the offspring, 73 chicks were hLY positive, including 40 females (S1 Fig). After that, G3 roosters were used to generate G4 chickens. PCR screening confirmed that 47 chicks were hLY positive among 90 detected progenies, including 25 females and 22 males (S1 Fig). The efficiency of transgene transmission between generations was consistent with Mendelian inheritance (Table 1).

**Table 1:** Numbers of transgenic chickens in generations of G3 and G4.

	Total number of offspring	Number of positive offspring	Positive rate
G3	139	73 (♀40)	0.53
G4	90	47 (♀25)	0.52

### Oviduct-specific expression of rhLY in G3 and G4 hens

**Fig 1:**
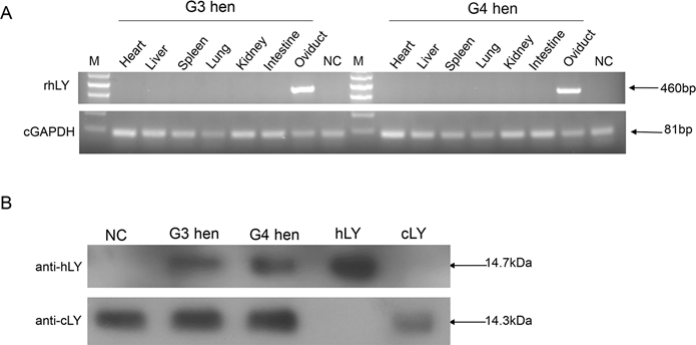
Expression of rHLY in transgenic chickens. (A) RT-PCR analysis of the expression of rhLY in G3 and G4 hens. Total RNA was isolated from the heart, liver, spleen, lung, kidney, intestine and oviduct of the G3 and G4 hens. NC (negative control) was the RNA of the oviduct from a non-transgenic hen. The 460bp fragments represented the RT-PCR products of hLY, and the 81bp fragments represented the RT-PCR products of GAPDH. M, DNA ladder. (B) Western blot of egg whites from a G3, a G4 and a non-transgenic hen (NC). Samples were separated by SDS-PAGE and hybridized with anti-hLY antibody and anti-cLY antibody, separately. hLY, commercial hLY (positive control for anti-hLY); cLY, commercial cLY (positive control of anti-cLY).

After G3 and G4 hens had been raised to sexual maturity, RT-PCR was first used to analyze the transgene expression in the heart, liver, spleen, lung, kidney, intestine and oviduct. The results demonstrated that hLY was specifically expressed in the oviduct (Fig 1A).

As it is necessary to make the recombinant proteins secret into the egg white, a signal peptide from cLY was added to the 5’ end of hLY [30]. Western blot demonstrated that rhLY was detected in the egg whites and expressed stably in both the G3 and G4 egg whites (Fig 1B). Quantitative analysis of Western blot revealed that the amount of the commercial hLY was about 1.3 times to that of the rhLY. Therefore, we concluded that the concentration of rhLY expressed in the transgenic egg whites was about 40 μg/ml. The hLY antibody did not detect the commercial cLY, and neither did the cLY antibody detect the commercial hLY. These results demonstrate that there are not any cross-reactions between the two antibodies (Fig 1B).

**Fig 2:**
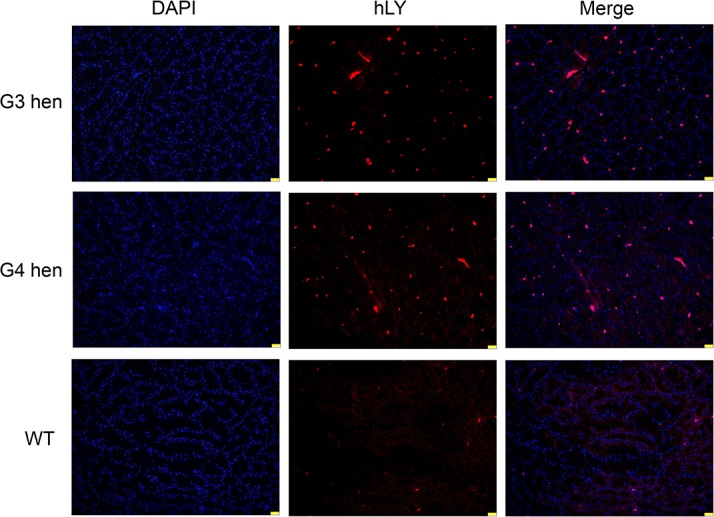
Immunofluorescence detection of hLY expression in oviduct sections. Sections of the magnum portions of the oviducts from a G3, a G4, and a wild type White Leghorn hen were immunolabeled with the anti-hLY antibody (red). The nuclei were stained with DAPI (blue). Scale bar: 25 μm.

Immunofluorescence on the magnum portions of the oviducts from a G3 and a G4 hen showed that rhLY was expressed specifically in the oviduct of transgenic hens, which agreed with RT-PCR results (Fig 2). In summary, rhLY was secreted into the egg whites, and the transgene transmitted to next generations without detectable silencing. This result is consistent with the research by Roslin Institute [42].

### Purification of rhLY from transgenic egg whites

**Fig 3:**
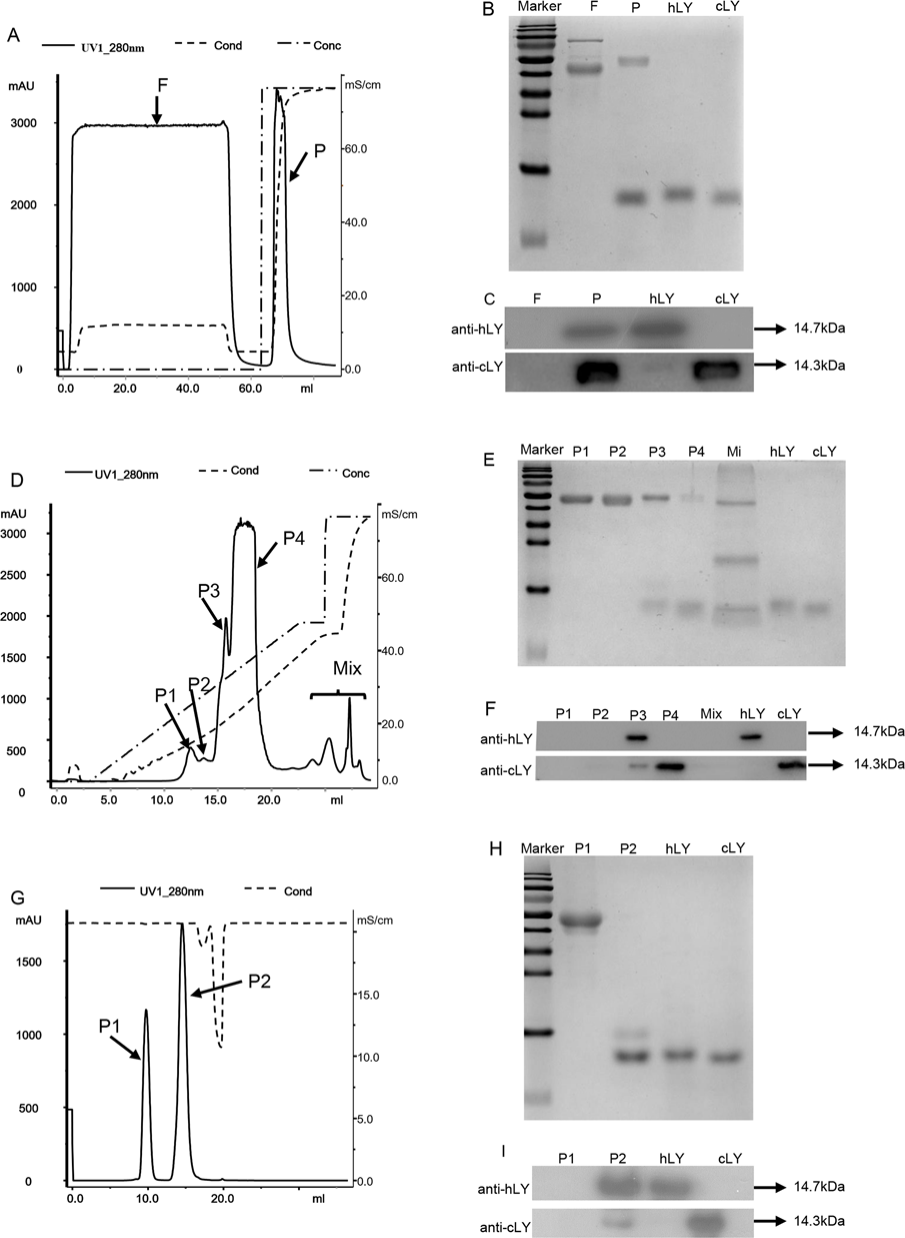
Purification of rhLY by cation-exchange chromatography and gel filtration chromatography. (A) Purification of rhLY by cation-exchange chromatography through the Hiscreen SP FF prepacked column. Lyophilized powder (20 g) from transgenic egg whites was dissolved in 20 mM sodium phosphate buffer (pH 8.5) and applied to the column. F, the flow through; P, the elution peak. (B) Examination of the flow-through and elution peak from the Hiscreen SP FF prepacked column by 15% SDS-PAGE and Coomassie blue. (C) Western blot of the flow-through and elution peak from the Hiscreen SP FF prepacked column. (D) Purification of rhLY from the first chromatographic step by the Mono S 5/50 GL prepacked column. The P fraction from the first step was loaded on the column after being desalted by 20 mM sodium phosphate buffer (pH 7.0). P1, P2, P3, P4 and Mix represented the elution peaks. (E) Examination of the elution peaks from the second chromatographic step by 15% SDS-PAGE and Coomassie blue staining. (F) Western blot of the elution peaks from the second step. (G) Gel-filtration chromatography of the partly purified rhLY from the second step through the Superdex^TM^ 75 10/300GL prepacked column. The P3 fraction from the second chromatographic step was applied to the column after being concentrated with an ultrafiltration centrifugal tube. P1 and P2 represented different elution peaks. (H) Examination of the rhLY from the third chromatographic step by 15% SDS-PAGE and Coomassie blue staining. (I) Western blot identification of the elution peaks from Gel-filtration chromatography. hLY, commercial hLY (positive control for anti-hLY); cLY, commercial cLY (positive control for anti-cLY).

The major constituent of egg white is acidic proteins, except for lysozyme and avidin [43]. According to this characteristic, we used a three-step chromatographic method to isolate rhLY from transgenic egg whites. After removing the bulk of ovomucin, a Hiscreen SP FF prepacked column was used in the first chromatographic step to collect the majority of alkaline proteins which were eluted with 1 M NaCl in 20 mM sodium phosphate buffer (pH8.5) (Fig 3A). Western blot results showed that cLY and rhLY were successfully collected in the eluent (Fig 3B and 3C). Because cLY and hLY share the similar characteristics and molecular weight, an effective method is needed to separate them. We next tested the Mono S 5/50 GL prepacked column with narrow particle size distribution in the separation of commercial cLY and hLY in a preliminary experiment. The result demonstrated that this method could distinguish the two proteins into two elution peaks (S2 Fig). Therefore, in the second chromatographic step, the eluents from the first step were loaded on the Mono S 5/50 GL column, and rhLY was further eluted with 0.4 M sodium chloride in 20 mM sodium phosphate buffer (Fig 3D). The rhLY was detected in the peak P3 by SDS-PAGE and Western blot analysis (Fig 3E and 3F). In the third chromatographic step, we applied gel-filtration chromatography and eluted the purified rhLY with 50 mM sodium phosphate buffer containing 0.15 M NaCl (pH 7.0). SDS-PAGE and Western blot showed that the high-purity rhLY was present in the peak P2 of the eluents (Fig 3G, 3H and 3I). Eventually, we obtained about 6 mg rhLY from 10 eggs (200ml egg whites). The purification efficiency was about 75%. Quantification of the final product with Image J demonstrated that the purified product contained more than 90% of rhLY and trace of cLY (Fig 3I).

### Analysis of rhLY properties: the molecular weight, peptide mass fingerprinting, and N-terminal sequence

The mass spectrometry result by 5800 MALDI-TOF/TOF showed that the molecular mass of rhLY (14679.43 Da) was only 5.24 Da greater than that of the commercial hLY, similar to the commercial hLY (Table 2). The peptide mass fingerprinting analysis demonstrated that the purified rhLY resembled hLY. N-terminal sequencing indicated rhLY did not carry any mutations (Table 2), and the signal peptide was incised correctly. These results confirmed that we had successfully expressed and purified rhLY, which possessed the similar properties with the commercial hLY.

**Table 2:** Compare the properties of the purified rhLY and commercial hLY.

	rhLY from transgenic chicken	Commercial hLY
Molecular mass	14679.43 Da	14674.19 Da^1^
N—terminal sequence	KVFERCELARTLKRL	KVFERCELARTLKRL^2^
Antibacterial activity (U/mg)	33532.9±139.7	34011.1±786.2

^1^The molecular mass of commercial hLY had been measured previously [39]

^2^N - terminal sequence of the commercial hLY referred to GeneBank databases.

### Antibacterial activity of rhLY

**Fig 4:**
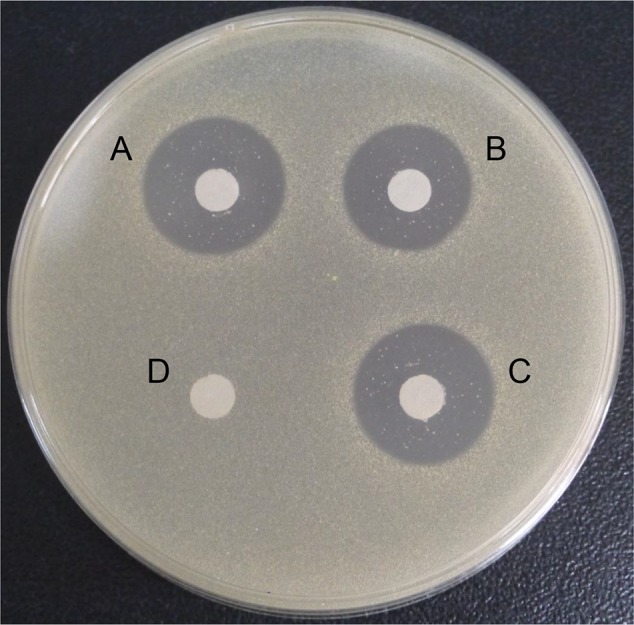
Antibacterial activity of rhLY, hLY, and cLY against *M*. *lysodeikticus*. The picture showed the inhibition zones of the agar disc diffusion of commercial hLY (A), commercial cLY (B), purified rhLY (C), and sterile water (D, negative control).

We used agar disc diffusion and turbidimetric method to analyze the lytic activity of the purified rhLY and compared it to commercial hLY and cLY. From the results of agar diffusion analysis, we could see that rhLY and commercial hLY showed the similar bactericidal activity, but both of them had the bigger inhibition zones compared with cLY. The sterile water was used as a negative control and did not exhibit any antibacterial activity (Fig 4).

The turbidimetric analysis further confirmed that the antibacterial activity of rhLY was similar to the commercial hLY and was about two to three times higher than cLY (Table 3). These results proved that we successfully purified rhLY with biological activities.

**Table 3:** Antibacterial activity of rhLY, hLY and cLY against *M*. *lysodeikticus* determined with the turbidimetric method.

	rhLY^2^	hLY^3^	cLY^4^
Concentration (mg/ml) ^1^	1.67	1	1
Activity (units/mg)	33532.9±139.7	34011.1±786.2	13977.8±830.2

^1^The concentration of rhLY was quantified by BCA Protein Assay Kit

^2^rhLY is purified rhLY from the egg whites of the transgenic chicken

^3^hLY is commercial hLY

^4^cLY is commercial cLY.

### Optimal working temperature and pH value of rhLY

**Fig 5:**
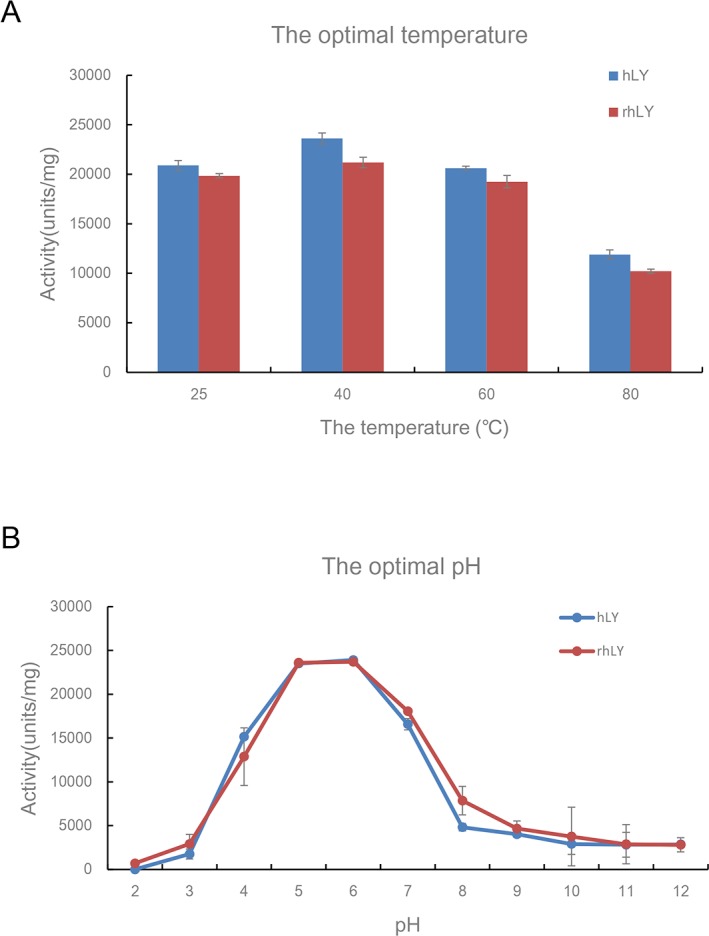
Optimal conditions of purified rhLY and hLY for their antibacterial activity. (A) The optimal temperatures of the rhLY and hLY against *M*. *lysodeikticus* were measured in phosphate buffered saline (pH 7.18) at 25°C, 40°C, 60°C, and 80°C respectively. (B) The optimal pH values of the rhLY and hLY for the antibacterial activity was measured in phosphate buffer with different pH values (from 2–12), separately. hLY, commercial hLY; rhLY, purified rhLY. The experiment for each group was repeated at least three times, and the results were presented as mean ± S.D.

We compared the optimal conditions for the bactericidal activity of the purified rhLY and commercial hLY. The results (Fig 5A) showed that these two proteins had the similar activities at different temperatures (P>0.05). Both of them showed higher activity at 40°C than other temperatures (P<0.05). The activity of the purified rhLY and commercial hLY at 25°C was not significantly different from that at 60°C (P = 0.20). Therefore, the optimal working temperature of lysozyme was 40°C.

As for the pH value analysis in phosphate buffer, in the buffer of pH 5 and pH 6 the rhLY and commercial hLY exhibited the highest lytic activity. Meanwhile, the difference between rhLY and hLY was not significant (P = 0.60) (Fig 5B).

### Thermostability and pH stability of rhLY

**Fig 6:**
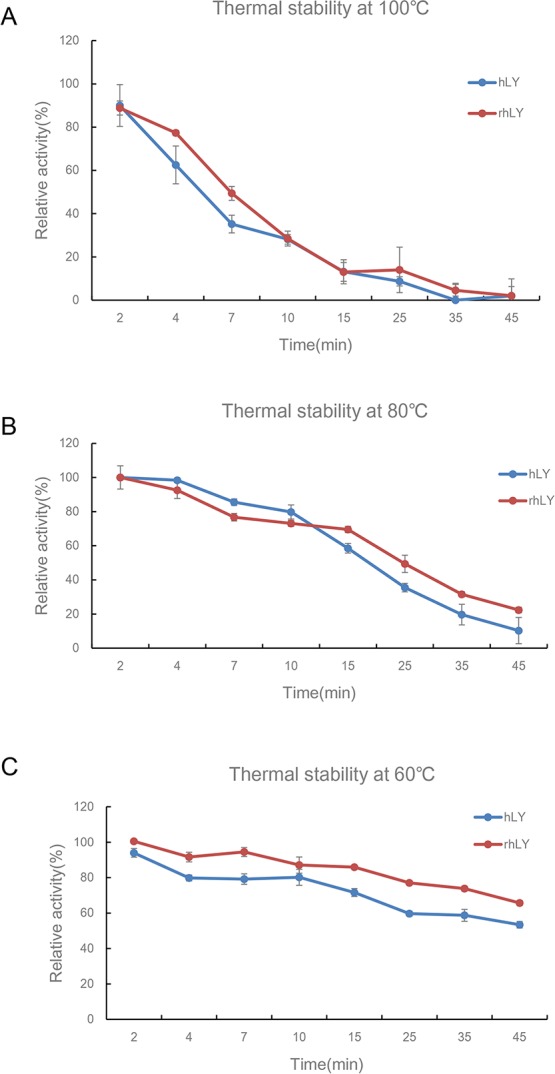
Thermostability of purified rhLY and hLY at 100°C, 80°C and 60°C. The lysozyme samples were first incubated for different periods of time at 100°C (A), 80°C (B), and 60°C (C), and their antibacterial activity against *M*. *lysodeikticus* was measured in phosphate buffered saline (pH 7.18) at room temperature. Lysozyme activity without heat treatment was defined as 100% activity. hLY, commercial hLY; rhLY, purified rhLY. The experiment for each group was repeated at least three times, and the results were presented as mean ± S.D.

We compared the tolerance of rhLY to that of the commercial hLY under high-temperature conditions. We found that both of them had the lowest thermal stability at 100°C (Fig 6A). Fifteen minutes of treatment at 100°C disrupted almost all the antibacterial activity of rhLY and commercial hLY. Under 80°C environment, rhLY could maintain about 60% of its activity for 15 minutes, and quickly lost the antibacterial activity after that (Fig 6B). The purified rhLY exhibited a higher tolerance at 60°C than the commercial hLY. After incubation for 45 minutes, it still kept 60% of its activity (Fig 6C). These results suggested the commercial hLY and rhLY exhibited the similar thermal stability.

**Fig 7:**
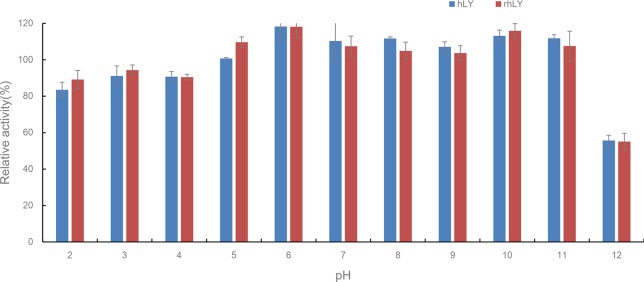
Stability of purified rhLY and hLY under different pH conditions. The lysozyme was incubated in phosphate buffer of different pH values (from 2–12) for 20 minutes. Then the antibacterial activity against *M*. *lysodeikticus* was measured in phosphate buffered saline (pH 7.18) at room temperature. The lysozyme activity incubated in phosphate buffer at pH 7 was defined as 100% activity. hLY, commercial hLY; rhLY, purified rhLY. The experiment for each group was repeated at least three times, and the results were presented as mean ± S.D.

For pH stability analysis, we found that both hLY and rhLY displayed an extensive tolerance with more than 80% of activity maintained under the conditions of pH2-11 (Fig 7). However, after a 20-minute incubation at pH 12, almost half of the lytic competence was lost.

## Discussion

The development of transgenic technologies enables the generation of transgenic animals for the production of recombinant proteins. The foreign proteins expressed by animal bioreactors could process posttranslational modifications, compared with the recombinant proteins synthesized in microbial cells [44]. The posttranslational modifications confer the recombinant protein biological activity and stability and make their purification easy. In addition, the population of transgenic animals could be expanded by conventional breeding, making the production of recombinant proteins cost effective. Moreover, using bioreactors to produce recombinant proteins could avoid industrial pollution. The oviduct bioreactor and mammary gland bioreactor have been thoroughly studied for many years. Comparatively, the oviduct bioreactor has many advantages, such as the short time for setup, the easiness of animal raising, the appropriate glycosylation of target proteins, and the high yield of the foreign proteins [28,29]. Moreover, the composition of egg white is simple, making it easy for recombinant protein purification.

In our previous work, Cao et al. used lentivirus vectors to generate transgenic chickens with the oviduct-specific expression of rhLY, and obtained four founders with different transgene insertion sites [30]. We used founder A011 whose insertion site is on Z chromosome for all further studies. Segregation analysis demonstrated a typical Mendelian inheritance of the transgene. RT-PCR and immunofluorescence analysis of G3 and G4 hens showed that rhLY was specifically expressed in the oviducts of transgenic chickens. The concentration of rhLY in the egg whites of G2 hens was about 45 μg/ml [30]. Western blot demonstrated that the expression level of rhLY in G3 and G4 hens was still about 40 μg/ml. These results demonstrated that rhLY transgene could stably transmit to next generations without being silenced. Our transgenic system was also used by Liu to generate transgenic chickens with the oviduct-specific expression of human neutrophil defensin 4 [31], another example proving that the transgene system was stable and feasible.

Arora et al. found that fresh egg albumen contained about 3 mg/ml chicken lysozyme [33]. Compared with cLY, the expression level of rhLY in our transgenic chickens (about 40 μg/ml) was still low. Previous studies demonstrated that higher rhLY expression was achieved in mouse mammary gland tissues (about 154 μg/ml) [45] and transgenic pig mammary glands (about 140 μg/ml) [19]. Both studies used the genomic DNA of hLY as the transgene construct rather than the cDNA construct used in our transgenic chickens, suggesting that the introns of the hLY gene could increase its transcription [46]. Additionally, Maga et al. used the 540 bp hLY cDNA driven and regulated by the 23 kb promoter and 3’ regulatory elements of the bovine α_S1_-casein gene to generate the transgenic goats. The expression level of hLY in the mammary gland was about 270μg/ml [26]. Jianmin Huang et al. used a construct containing the RAmy3D promoter, RAmy3D signal peptide coding sequences, and RAmy3D terminator to produce transgenic rice, and the expressed hLY made up to 2.5 or 4.2% of total soluble proteins in rice calli [47]. Different from our study, these two studies used the longer promoter sequences and more regulatory elements, which affected the expression of foreign proteins. We speculated that optimized regulatory elements could elevate the expression of transgenes.

Position effects could be another factor influencing the transgene expression. Lu and Yang used the same expression plasmid to produce transgenic pigs and cattle, separately, but Lu obtained higher hLY expression due to the different insertion sites from Yang’s study [19,39]. Liu also found different insertion sites affected the foreign gene expression [31]. Therefore, we conjectured that certain specific transgene integration positions would effectively increase the expression of foreign genes. The development of genetic modification of primordial germ cells (PGCs) [48–51] could help us achieve gene targeting in transgenic birds, and overcome the position effects. Knocking in the transgene at the specific position, such as the ovalbumin gene site in transgenic birds, could increase the production of the recombinant proteins effectively. Additionally, the modification of PGCs in vitro could also overcome the limited capacity of lentivirus vectors, which could provide more effective regulatory elements to improve the expression level of the foreign proteins.

Affinity chromatography and ion-exchange chromatography are commonly used to purify hLY. The later one owned many advantages, including easy manipulation, high efficiency, and low cost. Thus, the ion-exchange chromatography is the primary method to isolate hLY. Up to now, this method had been used to purify hLY from filamentous fungus *Aspergillus niger*, transgenic rice seed, and many other bioreactor systems [21,36,37,39]. Mono S is a strong cation exchanger with the narrow particle size distribution. It was used by Fang to isolate *Bauhinia purpurea* trypsin inhibitor from the seeds of *B*. *purpurea* [52], as well as by Ye to purify a 30kDa antifungal protein from red cabbage [53]. The use of Mono S overcame the interference from the similar proteins during the purification process. About 3.4% of albumen in eggs is cLY [43], which shares high homology in amino acids with hLY, and has the similar molecular weight to hLY. These characteristics interfered with the separation of rhLY from eggs. We demonstrate here that Mono S could separate hLY from cLY. We obtained about 6 mg rhLY with the purity exceeding 90% from ten transgenic eggs, and the purification efficiency was about 75%. The procedure we used contained two steps of cation-exchange chromatography and one step of gel-filtration chromatography, which is low-cost, highly efficient, and could be simply manipulated. More importantly, the final recombinant protein product could reach high purity and maintain its biological activity. Thus, it could be scaled up in the future to provide the foundation for industrial purification of recombinant proteins expressed in transgenic eggs.

Our analysis proved that the physical and chemical characteristics of the purified rhLY were similar to the commercial hLY. Although the molecular weight of rhLY was about 5 Da difference from the commercial hLY, it was in a reasonable range of possibly different post-translational modifications [54]. Peptide mass fingerprinting of rhLY revealed its similarity to hLY, which denoted that they had the same primary structure. N-terminal sequencing did not show any amino acid changes, indicating that the cLY signal peptide we used could be cut correctly, and the mature rhLY could be secreted into the magnum of the oviduct.

The antibacterial activity of rhLY against *M*. *lysodeikticus* (gram positive bacteria) was similar to that of the commercial hLY, which was three times higher than cLY. This demonstrated that the purified rhLY maintained high biological activities. The optimal working condition of rhLY was 40°C, pH5-6, which was same as the commercial hLY. The purified rhLY also exhibited higher tolerance at 60°C and maintained its biological activity at a wide range of pH values. Even in the strong alkaline condition (pH 12), rhLY remained about 50% of lytic activity for 20 minutes. As rhLY has more tolerance to high temperatures and a broad range of pH values, it could benefit the industrial production of rhLY.

In our study, we found that the rhLY transgene could transmit to next generations without being silenced. Moreover, we successfully isolated rhLY from the transgenic eggs through a three-step chromatographic procedure. The purified proteins maintained their biological activity and exhibited the similar physical and chemical characteristics as the commercial hLY. To sum up, our transgenic chickens could produce the functional rhLY, and the purification procedure could provide options for the separation of other recombinant proteins expressed in the egg whites.

## Supporting Information

S1 Fig Identification of G3 and G4 transgenic chickens by PCR. M, marker; PC (positive control), genomic DNA from G2 blood; NC (negative control), genomic DNA of non-transgenic chicken blood.
(TIF)

S2 Fig Isolate hLY from cLY with a Mono S 5/50 GL prepacked column. The commercial hLY and cLY were loaded on the column. After eluting with a linear gradient of 0–1 M NaCl in 20 mM sodium phosphate buffer (pH 7.0), two elution peaks represented hLY and cLY were separated.
(TIF)

S1 Table Sequences of primers used in the study. (DOCX)
