# Supplementary figures and images for: Purification and Characterization of Recombinant Human Lysozyme from Eggs of Transgenic Chickens
Source: PLoS One. 2015 Dec 29;10(12):e0146032. doi: 10.1371/journal.pone.0146032 (PMC4694923; doi:10.1371/journal.pone.0146032)

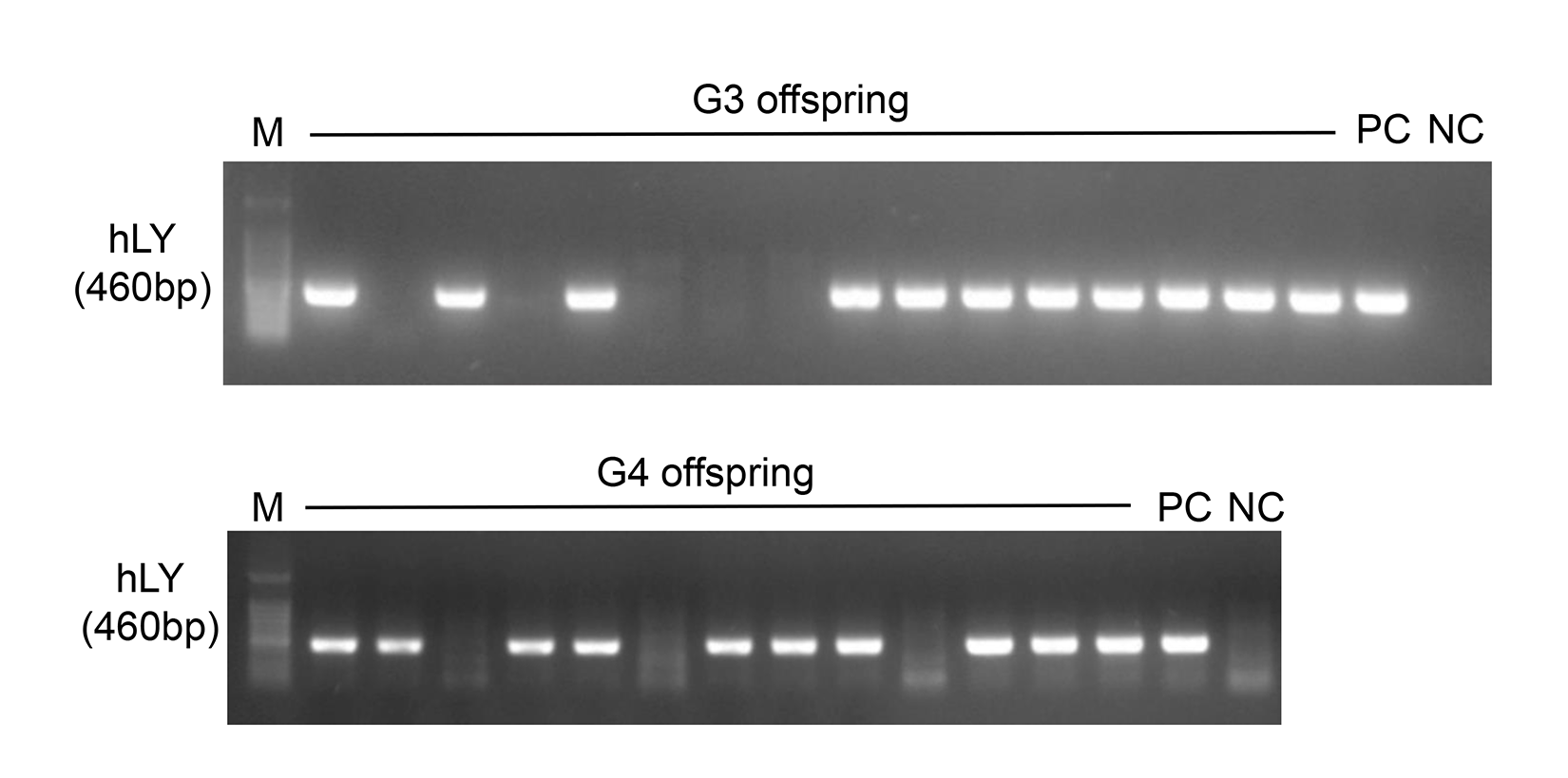

Supplement: S1 Fig — M, marker; PC (positive control), genomic DNA from G2 blood; NC (negative control), genomic DNA of non-transgenic chicken blood. (TIF) [file pone.0146032.s001.tif]

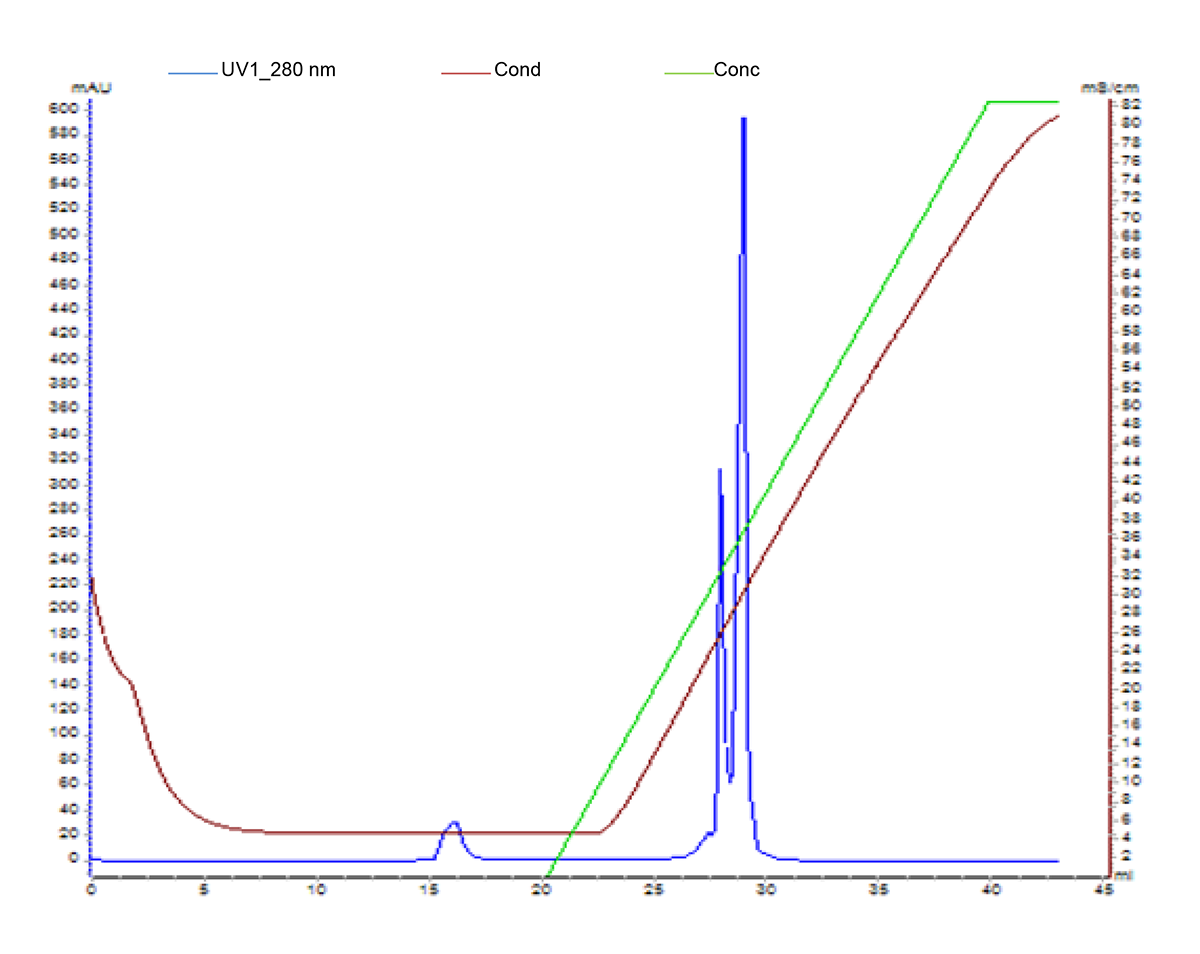

Supplement: S2 Fig — The commercial hLY and cLY were loaded on the column. After eluting with a linear gradient of 0–1 M NaCl in 20 mM sodium phosphate buffer (pH 7.0), two elution peaks represented hLY and cLY were separated. (TIF) [file pone.0146032.s002.tif]
