# Supplementary material for: Purification and Characterization of Recombinant Human Lysozyme from Eggs of Transgenic Chickens
Source: PLoS One. 2015 Dec 29;10(12):e0146032. doi: 10.1371/journal.pone.0146032 (PMC4694923; doi:10.1371/journal.pone.0146032)
Supplement: S1 Table — (DOCX) [file pone.0146032.s003.docx]

**S1 Table. Sequences of primers used in the study.**

| Primer | Sequence (5’-3’) |
| --- | --- |
| hLY-F | TGTACGACACTGGCAACATG |
| hLY-R | CACTCCACATCCCTGAACATA |
| sex-F | AGGATGGAAATGAGTGCATTG |
| sex-R | CATCTGGTGGTTTAATGAGGT |
| RT-GAPDH-F | CGATCTGAACTACATGGTTTACATGTT |
| RT-GAPDH-R | CCCGTTCTCAGCCTTGACA |
